# The Value of the Pathologist to the Surgeon

**Published:** 1911-06

**Authors:** E. C. Davis

**Affiliations:** Atlanta, Ga.


### THE VALUE OF THE PATHOLOGIST TO THE SUR-SURGEON.*

By Dr. E. C Davis, M.D., Atlanta, Ga.

The importance attached to co-operating in diagnosis as an essential to accuracy has been impressed upon me so many times, especially recently, that I believe the general profession should become better acquainted with the advantages which may accrue from the surgeons' experiences in connection with scientific pathological examinations.

Were it possible, no operation would be performed, except minor and emergency ones, without a preliminary careful examination of all the secretions and excretions, blood, etc. available, and, were such rules more rigidly enforced, together with the careful clinical examinations, there would be fewer errors and I dare say, in a few instances, a reduced mortality in surgical reports.

I am not in any way endeavoring to disparage the careful clinical examinations with that knowledge which is only acquired by careful, painstaking observations and oftimes numerous physical examinations, but rather to add to that the thorough, scientific laboratory methods presided over by a trained and competent pathologist. The true value of this could perhaps best be illustrated by a few cases which have recently been under my care.

Mrs. P., aged 42, had not passed menopause. Uterine hemorpause, reported recurrence of flow. On examination found slightly eroded cervix, small uterus and but little clinically to cause a positive diagnosis. She was given ether, uterus curretted and section of cervix removed. Scrapings and tissues were sent to a pathologist, with report rendered that we had a carcinoma of cervix. Abdominal hysterectomy was done, and patient appears well some months after operation.

Mrs. P., age 42, had not passed menopause. Uterine hemorrhage, similar treatment as above. First report benign. Patient was allowed to go home after curettage only to have recurrence of hemorrhage with clinical symptoms of malignancy. She was now subjected to a vaginal hysterectomy and uterus examined by pahologist, who reported a corporeal sarcoma of uterus.

Patient, clinic, Atlanta School of Medicine; hysterectomy for tumor; suspected multiple fibro-myomata. On microscopical examination this was found to be carcinoma.

I could give many of clinic cases which symptomaticaly and clinically appeared to be fibroids that proved later, on microscopical examination to be either incipient carcinoma or sarcomata.

Mrs. G., uterine hemorrage; had not passed menopause; had previously been curetted twice only to have a recurrence of the hemorrhage and when admitted to sanatorium, her blood only showed 30% hemaglobin. Under forced feeding, ferruginous tonics, living in open air, this was increased to 65% to 70% and a hysterectomy performed, removing uterus filled with small fibroids and completely relieving her of her discomforts.

Mrs. B., age 45; uterine hemorrhage, greatly fearing malignancy; uterus slightly enlarged and very suspicious to touch. Hysterectomy was performed, removing uterus which was submitted to a pathologist, who reported numerous small mural fibroids.

Mrs. A., chain of enlarged glands on both sides of neck simulating clinically Hodgkins disease. She was examined, her blood and tubercular tests made, pronouncing case tubercular adenitis. The tubercular lymphatics were removed and patient subsequently treated by Dr. Thrash for tubercular condition with great relief.

I could recite numerous instances where vaginal discharges were examined to determine the presence of gonococci. Some, where clinical signs were strongly suspicious, microscopic examinations proved negative.

The latter cases not alone were of interest from pathological complications that were eliminated, but also the domestic difficulties that were often avoided.

I could also recite cases of Leucocythemia coming for operation for suspected tumors of spleen, cleared up by examinations of blood.

Numerous cases of diabetis, mellitis appearing for operation, are only recognized by careful examination.

These cases could be added to almost become tiresome, but they are sufficient to prove that there should be a closer and more frequent association between the surgeon, the medical man and especially the pathologist. In fact, the time has now come when patients must be studied more scientifically, and to use a rather vernacular phrase, in so doing we must utilize "team work" to get the best results.

Scientific organization with the skilled man in charge of his respective department means more to-day than ever before.

Patients should if possible be under observations sufficiently long to determine more accurately conditions than has been previously done.

The recognition of the types of organisms occurring after the various infections and the preparation of the autogenous serums has proven of great value in a few cases.

The pathological reports have been made largely by Drs. Thrash and Bunce, to whom I feel much indebted.

